# Duloxetine and suicide attempts: a possible relation

**DOI:** 10.1186/1745-0179-4-18

**Published:** 2008-06-11

**Authors:** Bilal A Salem, Elie G Karam

**Affiliations:** 1 Department of Psychiatry and Clinical Psychology, Saint George Hospital University Medical Center, Beirut, Lebanon; 2 Department of Psychiatry and Clinical Psychology, Faculty of Medicine, Balamand University Medical School, Beirut, Lebanon; 3 Institute for Development, Research, Advocacy and Applied Care (IDRAAC), Beirut, Lebanon; 4 Medical Institute for Neuropsychological Disorders (MIND), Beirut, Lebanon

## Abstract

The possible increase of suicidal ideation with some antidepressants is still a matter of debate. We are reporting two cases of suicidal attempt that occurred concomitantly with the use of Duloxetine. To our knowledge there is no prior publication about a possible Duloxetine related increase in the risk of suicidality.

## Background

An increase in suicidal ideation with antidepressants is still a matter of debate [1-11]. The advent of SSRI antidepressants appears to have been accompanied with significant decrease in suicide rates in most countries with traditionally high baseline suicide rates [12,13]. More recent larger clinical studies, in both adults and children have confirmed the protective effects of antidepressants against suicidality. The increased use of antidepressants has reduced depressive morbidity and suicidal morality. The significant relation between increasing antidepressant utilization and decreasing national suicide rates does not automatically suggest a causal association, but considering the strong (and causal) relationship between untreated major mood disorder and suicidal behavior most pharmaco-epidemiological studies strongly suggest that more widespread and effective treatment of mood disorders is a significant, although not the only factor in decreasing suicide mortality at the level of the general population[14].

Many of the suicide attempters with depression have been thought to have "mixed" depressions with a 4.4 odds compared to non-mixed depressions [15]. Of the symptoms of mixed depression, there is a significant association between suicidal ideation, psychomotor activation and racing thoughts which bears support to the hypothesis that agitation is a suicide risk factor in patients with depression [12]. The issue remains whether the "activation"/"agitation" seen with the use of antidepressants is the direct results of their use and if this is the main factor in the observed association between their use and the possible increase in suicidality in some reports[15,16].

## Case reports

### CASE I

A thirty year old married female was transferred to SGUMC (Saint. George University Medical Center/Beirut) for psychiatric evaluation and treatment after she had attempted suicide by ingesting 250 ml of a detergent containing a fatal substance (HCL) which had caused a severe pyloric stenosis. History revealed an onset of major depressive disorder for which she had been put on Duloxetine 60 mg in addition to the Alprazolam she was already on. Five days after the initiation of Duloxetine, she felt an exacerbation of her depressive symptomatology with an emergence of new symptoms of irritability and agressivity. These symptoms worsened in severity and two weeks after starting Duloxetine, she attempted suicide in the context of extreme irritability. She had no history of previous suicidal attempts.

### CASE II

A forty seven year old married female was admitted to SGUMC for management of depression following a suicide attempt (5 weeks post an overdose of Alprazolam). She had a three year history of depression and generalized anxiety disorder with multiple somatic complaints and had been on Duloxetine 60 mg with Alprazolam 2 mg HS for two months during which she reported improvement in her mood as well as her somatic symptoms except for lower limbs pain. Duloxetine had been then increased to 120 mg daily. About five days after this increase the patient started feeling more pain with a new onset of irritability. The depressive symptoms and irritability kept on escalating and on the third week after the increase of Duloxetine, the patient attempted suicide with an overdose of Alprazolam (22 mgs) without any plan. The patient reported having a previous suicidal attempt at the age of fifteen years.

## Discussion

In both cases there is an emergence of suicidality in apparently nonsuicidal patients after starting or increasing Duloxetine. The duration after which suicidality emerged is similar to what has been reported in the literature i.e. 1–9 days [1-3]. In addition the symptoms that preceded the suicidal attempts readdress the issue of antidepressant induced suicidality occurring in the context of agitation, irritability and impulsivity [3,4,9,10]. Antidepressant monotherapy in patients with bipolar disorder and the bipolar spectrum could worsen the preexisting mixed state of depression or generate mixed conditions de novo, resulting in treatment resistance, destabilization of the mood disorder, worsening of the depression and, possibly suicidal behavior in some patients [12,16]. The recognition of the important role of pseudo-unipolar mixed states of depression in suicidal behavior has clear implications for suicide prevention. However, the rare occurrence of suicidality on antidepressants should not obscure the fact that the advent of the new antidepressants is associated with worldwide decline in suicide rates [13].

## Conclusion

Observations do not necessarily implicate antidepressants as being causative, but the possible emergence of suicidality could be mediated through what is referred to as the "activation syndrome" possibly in patients with potentially a bipolar spectrum. Clinical wisdom would suggest that, faced with depressed patients, one must exercise extreme caution in the first 4 weeks after prescribing antidepressants especially if unprotected by mood stabilizing agents and/or atypical antipsyhotics [16].

## Conflict of interests

The authors declare that they have no competing interests.

## Authors' contributions

BS participated in writing the case reports after gathering the needed information, formulating the cases and reviewing of the literature.

EK treated the two patients at SGUMC, participated in reviewing and writing the case reports, and doing part of the literature review.

## Funding

IDRAAC http://www.idraac.org for secretarial and logistic support.
